# Review on ''*Bioinformatics*, *Biocomputing and Perl*'' by Michael Moorhouse and Paul Barry

**DOI:** 10.1186/1475-925X-3-33

**Published:** 2004-10-12

**Authors:** Artem Cherkasov

**Affiliations:** 1 Division of Infectious Diseases, Faculty of Medicine, University of British Columbia. 2733, Heather street, Vancouver, British Columbia, V5Z 3J5, Canada

## 

The book "*Bioinformatics*, *Biocomputing and Perl*" [1] attempts to encompass those numerous volumes which most bioinformaticians keep on their office bookshelves and which are often entitled as "S*omething in a Nutshell*".

The book aims at both biology- and computation-oriented audiences and is designed as a number of 'crash-courses' quickly updating the reader on the basics of bioinformatics. It starts with a preface outlining main biological and technological concepts of the modern computational biology. The rest is organized into four sections consisting of 18 chapters elaborating on essential bioinformatics tools and skills.

The section 'Working with Perl' presents an extended tutorial with practical tips and useful references for Perl beginners. Following this is 'Working with Data', which familiarizes the reader with some public genomic and proteomic databases and discusses important subjects of database formats, non-redundancy, cross-referencing and programmable access, *etc*. By working through the section, the reader acquires basic skills for *mySQL *database use and DBI Perl programming.

Next, the authors offer Perl-based solutions for remote database access and for creation of WWW-based bioinformatics services using Perl functionalities in 'Working with the Web'.

The final topic of the book, 'Working with Applications', features basic tools for sequence alignment, protein homology modeling and data visualization, all commonly used in bioinformatics practice. The section also offers recent and relevant examples of *BioPerl *applications.

In general, the book reflects the state of bioinformatics field with its strengths and weaknesses. Many Perl chapters, such as Perl regular expressions, modular organization, DBI-programming, *BioPerl *and web-automation, are excellent. The presented material is rather comprehensive and yet easy to read – the authors spent appreciative efforts to make the book interesting and enjoyable. The authors also acknowledge the open-source nature of Perl and the bioinformatics community and offer on-line support and direct feedback to the readers.

There are also certain aspects, in which the book could be further improved. Several sections may be too advanced for the beginner level (such as Perl basics and database downloading), while others may contain too excessive details (the Protein Databank section). In addition, it may be of advantage to mention *AcePerl *[2], Perl-programmable access to the SRS as well as XML- [3] and distributed data processing by Perl. The book would greatly benefit from color illustrations. Several figures in the 'biological' sections are not very informative or readable (such as Figure 10.5), and one contains a critical error (Figure 1.1).

A very useful feature of the book is the use of maxims that highlight key points throughout the text. The authors also provide helpful technical comments where necessary and offer practical exercises at the end of each chapter. The book is concluded with six appendices covering the Linux basics, Perl installation, operators, on-line support and suggested reading materials which, in my mind, benefit the book tremendously.

Thus, the overall product, the "*Bioinformatics*, *Biocomputing and Perl*", serves well its purpose as an introductory textbook and a resource of reference materials for bioinformaticians.

## List of Abbreviations used

**AcePerl **– is a Perl interface for the AceDB – a popular object-oriented bioinformatics database.

**DBI Perl **– the primary interface for database programming by Perl.

**BioPerl **– a collection of Perl modules specifically designed for several most common bioinformatics tasks.

**XML **– Extensible Markup Language – a popular standard for documents containing structured information.

**SRS **– the Sequence Retrieval System – a popular relational database for bioinformatics.
